# Is Seeing Still Believing? Leveraging Deepfake Technology for Livestock Farming

**DOI:** 10.3389/fvets.2021.740253

**Published:** 2021-11-23

**Authors:** Suresh Neethirajan

**Affiliations:** Farmworx, Adaptation Physiology Group, Animal Sciences Department, Wageningen University and Research, Wageningen, Netherlands

**Keywords:** deepfake, animal welfare, animal emotions, artificial intelligence, livestock health, digital farming, animal based measures, emotion modeling

## Abstract

Deepfake technologies are known for the creation of forged celebrity pornography, face and voice swaps, and other fake media content. Despite the negative connotations the technology bears, the underlying machine learning algorithms have a huge potential that could be applied to not just digital media, but also to medicine, biology, affective science, and agriculture, just to name a few. Due to the ability to generate big datasets based on real data distributions, deepfake could also be used to positively impact non-human animals such as livestock. Generated data using Generative Adversarial Networks, one of the algorithms that deepfake is based on, could be used to train models to accurately identify and monitor animal health and emotions. Through data augmentation, using digital twins, and maybe even displaying digital conspecifics (digital avatars or metaverse) where social interactions are enhanced, deepfake technologies have the potential to increase animal health, emotionality, sociality, animal-human and animal-computer interactions and thereby productivity, and sustainability of the farming industry. The interactive 3D avatars and the digital twins of farm animals enabled by deepfake technology offers a timely and essential way in the digital transformation toward exploring the subtle nuances of animal behavior and cognition in enhancing farm animal welfare. Without offering conclusive remarks, the presented mini review is exploratory in nature due to the nascent stages of the deepfake technology.

## Introduction

Videos of politicians appearing to make statements they have never said in real-life, edited (revenge) pornography of celebrities, and movies with actors that have already passed away—deepfake technologies keep appearing in many different types of media, often while the audience is unaware of it. The term deepfake stems from combining the words “deep learning” and “fake,” as the technology relies on machine learning technologies to create forged content. Deepfake is a type of technology based on artificial intelligence (AI) that allows fake pictures, videos or other forms of media to be created through swapping faces or voices, for example. Popularly, deepfakes carry a tainted representation due to their adverse misuses that can result in manipulation, misinterpretation, or malicious effects. However, the technologies behind it, in particular the Generative Adversarial Networks (GANs), have a handful of advantages when it comes to biomedical and behavioral applications, and can even reach uses beyond humans. The creative algorithms behind this booming technology allow big datasets to be generated and can level up AI technologies to e.g., identify emotions, behaviors and intentions, and subsequently to predict them timely. This therefore opens up the possibility to be applied to a broad scientific audience, including but not limited to animal science. With an ever-growing population size, the demand for livestock continues to increase, raising numerous concerns about its environmental impact, animal welfare and productivity. In this article, I explain the basics of deepfake technologies, its (mis) uses and how it bears the potential to be applied to agricultural practices such as livestock farming.

## What is Deepfake and how Does it Work?

Deepfake, just like other deep-learning algorithms, rely on neural networks which simply said, is a software construction that attempts to mimic the functioning of the human brain. Deepfakes require source data samples, and an encoder and decoder. A universal encoder is used to analyze and compare the key features of the source data, which can be an image, video, text or audio file. The data are broken down to a lower dimensional latent space and the encoder gets trained to find patterns. The decoder is a trained algorithm that uses the specifications of the target to then compare and contrast the two images. As a result, the algorithm superimposes the traits of the source onto the image of the target resulting in the forged data.

**Figure 1:**
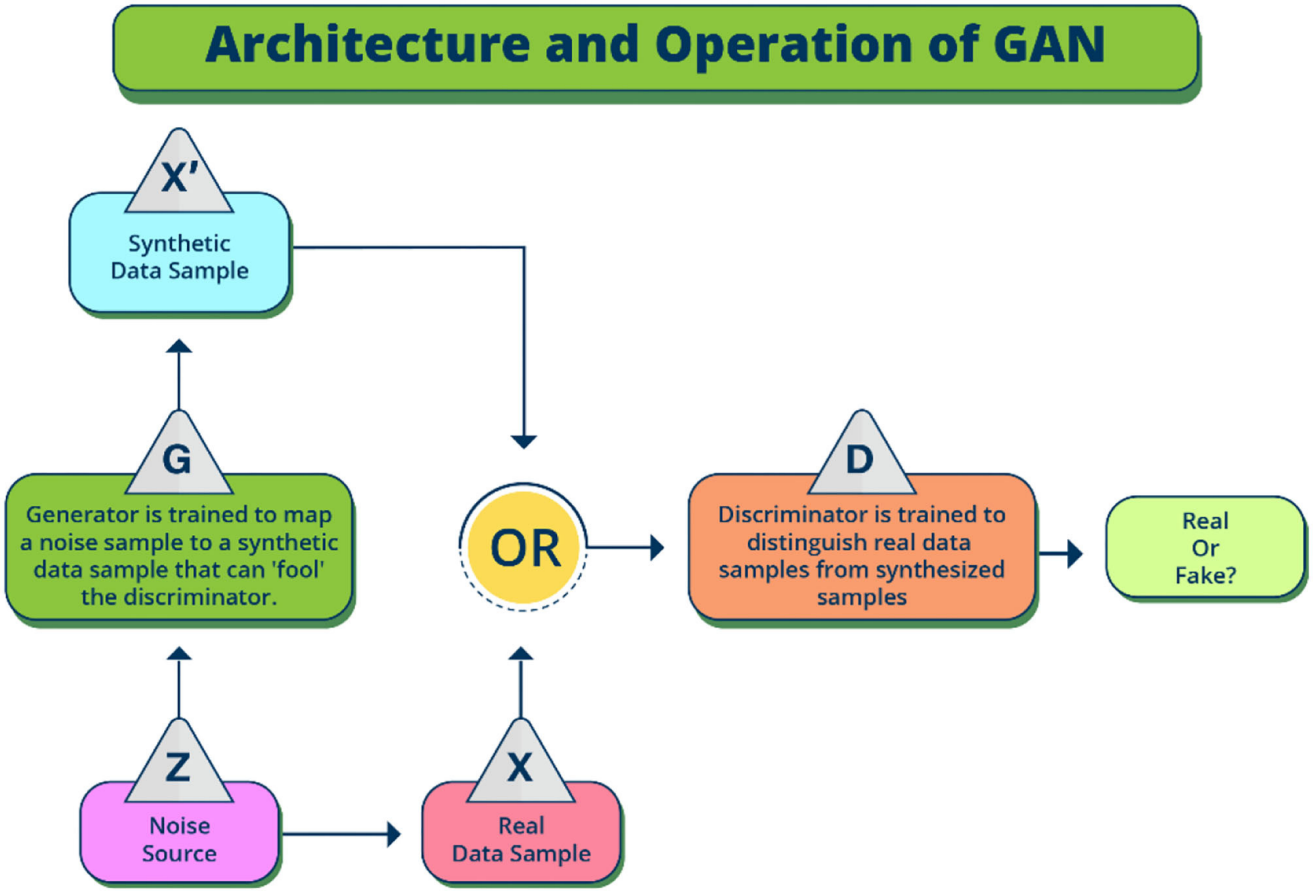
High level description of the Generative Adversarial Network (GAN) flow architecture. Real data sample is the source, while the synthetic data sample is the target. Input noise is combined with the label in the GAN architecture, and the framework of GAN training allows the conditional GAN loss function in the generation of synthetic target images.

The main architecture that allows a high precision and functioning of deepfake technology is the generative adversarial network (GAN) which is part of the decoder (1). Generally, encoder is employed in the extraction of latent features of faces or region of interest from images, while decoder is used in the reconstruction of faces. In the process of swapping faces between the target and the source image while creating the deepfakes, two pairs of decoder and encoder would be required, where each is first trained on the source and then on the target image. What makes GANs so unique and accurate is the operating and working together of the generator and discriminator. The generator creates a new image from the latent representation of the source data (Figure 1). The discriminator on the other hand tries to distinguish between the newly generated and the original real data as accurately as possible and determines whether the image is generated or not. As both networks perform adversarial learning to optimize their goals based on their loss function, the generator and discriminator continue to work together to constantly improve its accuracy. The applicability is highly powerful due to the continuous performance improvements and vector arithmetic in latent space. Moreover, GANs can create new datasets with a similar distribution and statistics as the main dataset used to train the algorithm. The discriminator learns about the distribution of the data, resulting in a model that can output new, realistic samples.

Deepfake technologies have been used to create software's and applications that generate fake images, texts or videos. Examples of these are apps that reproduce text with someone else's handwriting (“My text in your handwriting”), perform face swaps between humans but also from human to animals (“FakeApp”) and synthesize human voices (“Lyrebird”), amongst others. Open-source software's allow these technologies to be readily available to the public. Even though to date, it is still relatively intuitive to distinguish between real and fake, this distinction will start to fade as the technology advances. This development will increase the chance of misuse, manipulation, misinterpretation and spreading of fake news. Deepfake applications have therefore had a negative image due to the fear what may happen when falling in the wrong hands, to for example spread false information, pretending to be someone else or commit fraud.

However, the applications of deepfake technologies are not limited to (social) media purposes. The GAN model provides a sophisticated neural network with the big advantage that it can generate data based on a smaller, initial, real dataset. These frameworks have widespread uses, within fields such as biomedicine, behavior, affective science, but also beyond human applications.

## Using Deepfakes and Gans to Create Value

Whereas the negative applications of deepfakes and GANs can be scary, there are many positive ways to apply these models to create value for numerous fields of science that in turn, benefit humans and society. First of all, GANs are proving their high value in medical settings, such as to (1) recognize pathogens (2), (2) support a better and more effective screening and diagnosing of disease and abnormalities due to complementing MRI and CT imagery (3, 4) and (3) predict the progress of disease (5). Moreover, research within medicine can be facilitated through creating synthetic patient data that not only benefits the scarcity of medical data sets through replicating real-like data (4), but it can also be efficiently used for sharing, research, and in deciding treatment protocols and targeted interventions without needing to worry about patient privacy (6). In addition to this, mental health of clinical patients can be addressed through creative solutions using deepfake. For example, human subjects whom have lost their own voice, such as ALS (amyotrophic lateral sclerosis) patients, can be regenerated with GANs by using recordings of their original voice. Their own voice can then be used to communicate, instead of a generic computer voice synthesizer, to give the patients back a part of their identity (7). Outside the context of medical applications, GAN can also be used as classifiers to detect and classify the subject's emotional response (8). It can be beneficial for a plethora of applications, including patient health monitoring, crowd behavior tracking, predicting demographics (9) and similar behavioral applications.

But the potential applications of GANs are not limited to humans. Biologists, ecologists and ethologists are starting to understand the limitless applications of GANs especially in settings where obtaining high quantity and quality of data are difficult or impossible. Using these networks, scientists from different disciplines are starting to explore methods to e.g., simulate the evolutionary arms race between the camouflage of a prey and predator (10), to automatically identify weeds in order to improve productivity within agriculture (11) and to augment deep-sea biological images (12). These studies highlight the possibilities of GANs and lead to the possibility of using these technologies within livestock farming, too.

## Uses Beyond Humans—how Gans can Contribute to Increase Welfare in Livestock

As the global population is exponentially growing, it has been predicted that within a few decades, the demand for animal products will have doubled (13). This therefore puts a great pressure on the farming industry, that will need to keep up with the rising demand. The challenge to develop efficient processes of livestock farming is accompanied by a rising concern for animal health and welfare (14), in addition to environmental and societal concerns (15). Can GANs contribute to increase welfare in livestock, and as a consequence increase productivity, too?

Machine learning applications in animal science and the veterinary sector are predominantly focused on tracking activity and movement of the animals aimed at enhancing welfare or disease related measurements. In order to be able to use machine learning algorithms to, for example, automatically monitor animal health and welfare by screening and recognizing pain, stress and discomfort, large validated and annotated datasets are required. Physiological and behavioral measurements are able to reveal information about an animal's inner state. Animal emotions have been linked to particular vocalizations (16, 17), eye temperature (18, 19), hormone levels (20, 21) and facial expressions (20, 22, 23). These emotional states, such as fear, stress but also positive emotions like joy and happiness, remain however difficult to understand as they are complex and multi-modal.

AI algorithms can provide an automated way of monitoring animal health and emotions (24). This helps us understand animal behavior and stress that therefore can increase welfare by controlling and preventing disease and can increase productivity through helping famers decide on effective and productive strategies. However, validated and annotated datasets that are large enough for supervised machine learning algorithms are, however, limited and largely unavailable. Examples of specific medical conditions of farm animals and the related videos or animals are hard to come by and often require specialized sensing platforms and tools to collect. Due to this challenge, the advancements of applications of AI are still in the nascent stages in the farm animal sector. Supervised learning offers techniques to learn predictive models only from observations and maps an input to output by inferring a function from labeled data. Semi-supervised learning is concerned with using both labeled and unlabeled data to perform various learning tasks. Semi-supervised learning is a combination of unsupervised and supervised learning and uses a small amount of labeled data with larger unlabeled data (25).

There are a few methods to overcome the lack of high quality, labeled data. Semi-supervised learning helps *in situations* in which a large dataset is available but only a small portion of the dataset is labeled. Here, the challenge of insufficient datasets can be overcome by data augmentation methods. For example, augmentation techniques can include transformations such as translations by moving the image to left, right, up or down, by scaling such as zooming in or out, or by rotating the image to various degrees. Such techniques can help to expand the dataset size and is commonly used by data scientists for the data hungry ML models. But this standard method of enriching the dataset has several disadvantages; the produced images does not diverge far from the original image and may not add many varieties to enable the ML model or the algorithm to learn to generalize.

GANs have the potential to be used for enhancing the performance of the classification of algorithms in a semi-supervised setting, and it can address some of the barriers mentioned above. Training a GAN model has been successfully shown in augmenting a smaller dataset (2), such as for liver cancer diagnostic applications (26). It should be emphasized that the GAN based synthetic augmentation which uses transfer and deep learning approach is different than the basic (classical) data augmentation mentioned above. By adjusting the dimensions of the hidden layers and the output from the generator as well as input to the discriminator network, the framework was developed to produce satisfactory images of liver from the model. An accuracy of 85% was achieved by the GAN-created models in the liver lesion classification based on this method. In a similar way, GAN based data augmentation can be used to enhance the ability to classify animal disease and negative emotions such as stress and discomfort, that might lead to disease. A trained GAN model has the potential to predict diseases in farm animals and to recognize and avoid negative emotions such as stress and fear and promote positive ones. By creating bigger datasets with GANs with a similar distribution as the original datasets, machine learning algorithms could be trained to classify disease and animal emotional states accurately and efficiently, similarly as to how human emotions can be recognized by GAN models (8, 24, 27).

**Figure 2:**
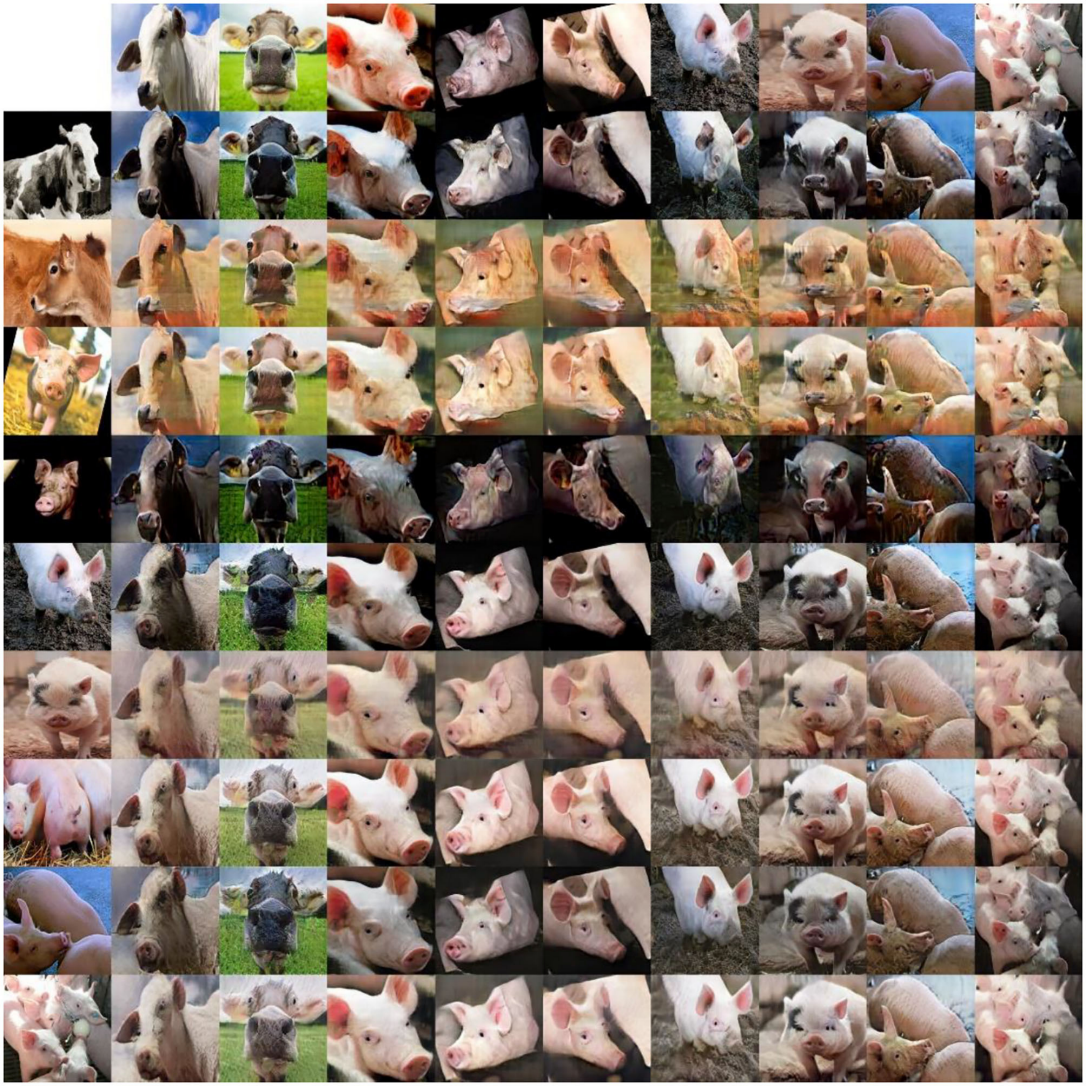
Example application of Generative Adversarial Networks (GAN) in livestock farming. Images of faces of farm animals such as cows and pigs generated by several epochs by the StarGAN architecture-based model. Quantitative comparison of the cow/pig dataset trained model is represented in each row. The real-life cow and pig are depicted in the top first row and the left first column. The StarGAN translated the source images of cows and pigs into target domains, reflecting the styles of the reference images as a precursor for the development of digital avatar of farm animals (36).

In addition to creating big fake datasets for classification, GANs could also be used to develop digital twins (28). A digital twin is a virtual representation of a real-world entity, such as a human or other animal. Based on input from the real world, the digital twin simulates the physical and biological state, as well as the behavior of the real-world entity. A digital twin of a farm animal will allow continuous monitoring of the mental, physical, and emotional state of the animals. In addition, modeling, simulating and augmenting the data allows the digital twin to be used to plan, monitor, control and optimize cost-, labor- and energy-efficient animal husbandry processes based on real-life data (29, 30). As a pre-cursor for the development of digital twin (digital avatar) of a farm animal, our group at Wageningen University has developed a methodology enabled by starGAN architecture in the generation of images of faces of cows and pigs (Figure 2). Using GANs to develop a digital twin will allow different situations to be explored and will help predicting its effects on the animals. It can, for example, be used to simulate and predict the effect of different housing structures or conditions, heat cycles for breeding or social settings on the positive and/or negative emotions of the animals, as well as on their productivity. Simulating different situations through digital twins will enable farmers to control and optimize processes within their operation, benefitting farming productivity, sustainability and animal health and welfare. Deepfake technologies can also offer a suitable non-animal alternative for biomedical research in the quest for provision of safe and effective drugs and treatments for both animals and humans.

Deepfakes (or virtual stimuli) have been suggested to help humans dealing with grief, by creating a virtual representation of the missing beloved (31). A similar approach could be taken to enhance animal welfare. Many farm animals are highly social, meaning that social comfort can play a large role in the mental wellbeing of the animals, but also that the maintenance of social organization is important for the entire population (32). The unnatural, monotone, high population-density setting of animal farms where animals are often regrouped and young are separated early from their mothers, can have adverse effects on their behavior and/or welfare (33). These effects range from stereotypies to high levels of (social) anxiety in early and later-life, and undesired behavior such as aggression that leads to conflict (e.g., tail biting in pigs, feather pecking in chickens) (34). Deepfake technologies can allow the display of videos of a (familiar) conspecific that simulates a companion, parent and/or dominant leader that brings back social organization which could serve as a tool to help fixing animal behavioral problems and in turn, enhance animal welfare. The interactions between an animal and its environment, including both conspecifics and humans are important to qualify and quantify. The GAN in combination with Machine Learning algorithms can learn about the different modes of animal communication that are important for the well-being of an individual, such as using facial expressions, vocalizations and body posture. Such features can aid in comforting one another and promote positive affective engagement with each other including affiliative interactions, sexual activity, bonding, maternal care and play behavior. These positive animal-to-animal interactive behaviors have been shown to play an important role in the positive welfare of (farm) animals (35). The trained model can then be used to optimize the digital representation in the form of e.g., a video that imitates such engagement, for example to assure young calves, chicks or piglets by a fabricated “mother” figure which aids a healthy development.

## Can Feelings of Farm Animals be Virtual? Emotion Elicitation in Digital Avatars

Exploring emotions in farm animals is very complex but a growing area of research. Researchers at Wageningen (https://farmworx.nl) in collaboration with ethologists and animal behavior scientists have been investigating the cognition and the behavior of the farm animals and thereby study the emotions of livestock. Typically, neuroendocrine, and hormonal markers such as dopamine, cortisol, lactate etc. are measured from the urine, saliva, blood, and hair of the farm animals in the cross validation for emotional indicators experiments. Several tests namely judgement bias tests, cognition experiments have also been developed to study negative, positive, and neutral emotional states of farm animals. A facial recognition system was recently developed to be able to measure and understand the manifestations of emotional expressions on the faces of cows and pigs (24). In addition, from our group and from other research groups worldwide it has been demonstrated that a number of non-invasive indicators such as respiration rate, heart rate, body surface temperature variations and other bodily behavior cues can present information on the emotional (affective states) makeup of the animals.

In the journey of developing “Life-like Agents/Metaverse/Digital Avatars” with the noble intention of solving practical problems in animal welfare, it becomes essential to establish frameworks for farm animal emotional modeling. Through integrating models of emotions and features or personalities of individual farm animals, the process of development of Digital Avatars can become easier. Context sensitive and purpose-based features of emotional patterning in humans have been explored as a theoretical model for creating autonomous emotional systems (37). In order to facilitate the development of life like artificial agents to generate emotions of their own, multiple computational models based on appraisal theory of emotions have been explored for human biomedical applications (38). Using a set of numerical values *via* computation rules, emotions has also been modeled as parameters of the agent for social simulation (39). Currently research is underway from our Farmworx research group in developing multimodal approaches-based emotion modeling for social interactions in cows and pigs. Deep fake technologies development in animals especially farm animals is in the nascent phase and hence no efforts has been made in emotional modeling for digital twins yet. However, with the advent of methodological frameworks being established in humans and the inspirations from the advancements of human affective computing, this gap in the farm animal emotional modeling will be addressed sooner.

## Voice Manipulator Produces Speech from Text

There is a definitive need for developing automated vocalization detection and reader systems for farm animals to enhance welfare (17). Vocalizations of animals such as cows, pigs and chickens can be in real-time translated to easily understandable text for animal caretakers and farmers to perform on the spot interventions. By taking the digitized audio recording and through processing and altering it *via* AI enabled algorithms, the sounds of farm animals such as grunts, squeals, coughing, sneezing, rooting, barking, panting can be measured and read continuously and non-invasively. In addition, the link between the emotions or affective states of farm animals and their vocalizations can be elucidated with the aid of deepfake enabled technologies. In this endeavor, fundamental research has been explored by application of computational methods in projecting animal vocalizations into latent representational spaces for visualization, characterization, and generation of signals in the investigations of ecologically relevant acoustic features (40, 41). Although the above-mentioned studies do not include livestock, but the vocalization has been explored only in bats and songbirds, the findings and the developed methodologies sets the path for future exploration of voice manipulation through deepfake approaches in domesticated production animals.

An advantage of using deepfake technologies is that other non-human animals, too, can be individually identified through their voice (42–44). Deepfake technologies that can base the generated data on a small fragment of the vocalization of an individual's mother, for example, will therefore be able to create a realistic mother figure rather than a general vocal sample. Outside of the mother-offspring context, vocal contagion of (positive) emotions can also be positively reinforced using the same technologies. The affective state of individuals can be influenced by its environment, and the literature shows that non-human animals can be affected by not only conspecific vocal expression of emotion, but also by human vocal expressions (45). This opens up the potential for deepfake technologies to positively influence farm animals through emotional contagion, promoting positive emotions.

Indirect evidence of discrimination and social recognition capabilities of farm animals and livestock has been investigated before. Examples include heifers' ability to visually discriminate their own species from other species (46); sheep recognizing unfamiliar and familiar human faces from 2D images (47) and female horses demonstrating the long-term memory by identifying the keeper from photographs after 6 months (48). Cattle use their sense of vision in the discrimination of conspecifics and demonstrated their ability to visually discriminate between familiar and unfamiliar conspecifics which were represented as 2D images (49). Moreover, with the rapid advancement of digital farming in which farmers have to be less present with the animals, also displays of positive interactions by “fake” farmers can be used to improve animal welfare. Such positive interactions could be used to reward good behavior, comfort the animals by reducing stress which in turn, have the potential to avoid unwanted behavior. These virtual farmer activities can therefore promote habituation, associative learning, social cognition and bonding, which could also enhance the human-animal relationship which is important for positive welfare outcomes as well as productivity (50).

**Figure 3:**
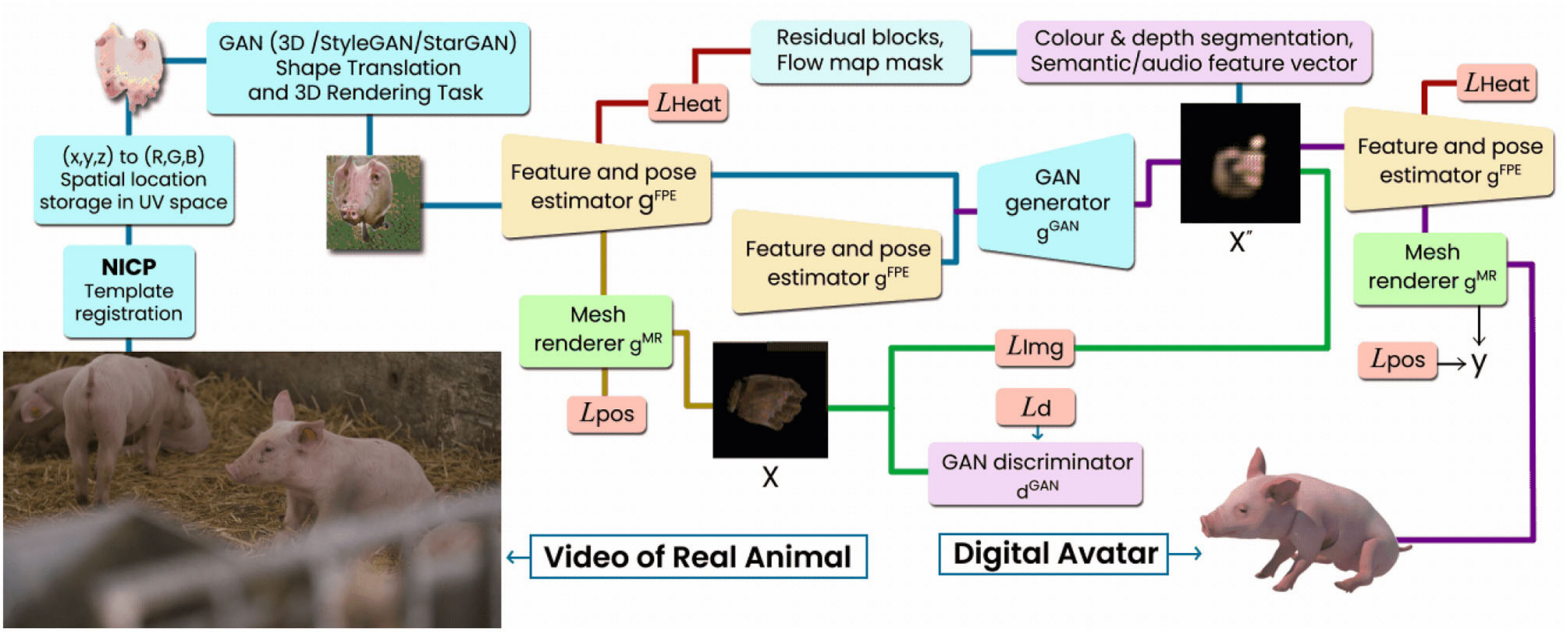
3D farm animal avatar digitization pipeline from real life video of a pig. Reconstruction of the geometry and texture *via* GAN model is followed by the generation of the full textured life like animal agent.

A video, of course, is merely a digital visual and maybe auditory representation of this conspecific, meaning that the physical and olfactory components of the virtual conspecific are lacking, which might limit its effectiveness. A better understanding of the cognitive framework and awareness of farm animals (51), and inter-specific differences between cognitive abilities are important to understand the potential effectiveness of 2D digital representations. It is essential to understand what cues are important to create a realistic virtual animal, and what senses are used to process the information. Future technologies might even develop 3D robotics using a combination of AI technologies including deepfakes, that could create a more realistic representation of another individual. Interactive systems based on advanced technological systems keep growing within domestic animal farms. Deepfake technologies can aid the development of animal welfare technologies through supporting interaction, activity, and sociality, putting the focus of the farm on its animals, their well-being and enriching activities. Figure 3 shows a research path in the development of digital avatar of farm animal based on GAN from real-life video of a pig. Exploratory experimental studies are required to test the effects of introducing a virtual conspecific and/or a sophisticated robot to enhance mental well-being and sociality in farm animals.

## Types of Gans—Which One is More Suitable than Others?

With the invention of GAN in 2014 (52), the generative models are becoming not only popular with several research applications but also showing impressive results in integrating audio, visual and text for large number of practical use case scenarios. Because of the success in the vision, several formulations of GAN namely StyleGAN, CycleGAN, pixelGAN, DiscoGAN, IsGAN and many more have been developed by researchers. It is not possible to objectively evaluate the progress of the training and the quality of the model developed by the GAN due to lack of objective loss function. Hence, formulation and the choice of GAN can be evaluated based on the output quality of the generated synthetic images or videos. In addition to inspecting the generated synthetic videos and images manually, Frechet Inception distance (FID) and inception score are some quantitative ways (53, 54) to assess the robustness of the GAN models.

## What Needs to be Done to Facilitate Deepfake Research and What are the Limitations that Need to be Addressed?

In order for deepfake technologies and their applications to be fully explored, it is important that the negative stigma on the technology are addressed first. See Table 1 for a summary of current and potential applications of deepfake technologies, both positive and negative ones. Many people are hesitant and scared due to the immense implications fake media can have when used to manipulate, misinterpret or abuse (55). The legal framework has yet to catch up with the proliferation of deepfakes. However, a comprehensive legal framework, if developed, would enable the deepfake recognition software to outcompete deepfake media creation, in ensuring that fake can always be recognized from real. Next, creative solutions for a range of different fields of science should be promoted to change the negative outlook on deepfake applications and highlight the positive uses of the yet relatively unexplored possibilities it opens up. Regardless of the particular application, it is important to not only have a recognized and well-established legal framework, but also an ethical one. The inherent nature of deepfake technologies is to create fake content, which is then used to deceive either humans, animals or machine learning algorithms. The ethical consequences have to be addressed by professionals from different disciplines to allow a broad understanding of the consequences of using deepfake.

**Table 1:** Summary of current and potential applications of GANs and deepfake technologies.

**Application**	**Positive or negative?**	**Explored yet?**	**References**
(Revenge) celebrity pornography	Negative	Yes	(56)
Spreading fake news	Negative	Yes	(57)
Creative editing for entertainment	Positive	Yes	(58)
Recreating handwriting and/or voices	Positive or negative	Yes	(59)
Manipulating images	Positive or negative	Yes	(60)
Human disease identifying, monitoring, and predicting progress; diagnostic information preservation	Positive	Initial stages	(61)
Farm animal disease identifying, monitoring, and predicting progress	Positive	No	–
Data augmentation for machine learning for low quality or quantity images	Positive	Initial stages	(62)
Data augmentation for machine learning in livestock farming	Positive	Initial stages	(31)
Improving therapy—Cyberpsychology	Positive	Initial stages	(63)
Identification and classification of weed species in agriculture	Positive	Initial stages	(11)
Identification and classification of animal emotions	Positive	No	–
Creating digital twins to monitor behavior and physiology of farm animals	Positive	Only in theory	–
Creating virtual conspecifics to increase mental well-being of farm animals	Positive	No	–

## Analysis of Potential Failures of Deepfakes for Livestock Farming

The biggest challenge is that the animal may perceive as presenting itself in the 'fake' world but in fact, the animal is still very much physically available in the real farm. The possibilities of the inability of the farm animal to distinguish between Digital Avatar and a 'real' flesh-based animal may lead to behavioral issues such as isolation or lack of adequate social interactions with other species. It may be possible that the farm animals may experience cybersickness (due to eye strain, dizziness) manifesting in the form of physical health or behavioral variations while engaging with digital avatars. Although this is an unexplored territory in livestock research, it is possible to overcome cybersickness due to deepfakes by manipulating the frame rates and refresh ratio while presenting for smooth engagement. Animal's living environment and the infrastructure such as the stable or industrial production facility or indoor farms should be accounted for while designing and developing the digital avatars.

Potential of farm animals colliding with farm structures or walls or even humans such as animal caretakers during the interaction with metaverse has to be considered. This could be due to the digital avatar reacting to the live animal or responding with exaggerated movement. One way to avoid the collision is by allowing the design features to consider developing complicated boundary spaces for the animals to interact with. This way, a trigger might induce the awareness of the presence of boundary and prevent the animal to go out of the boundary. Additional research is warranted to overcome the barriers associated with depth perception while designing deepfakes. Because farming environment and stable are dynamic and composed of full of structures, feeding stations and machinery, design factors must look at ways to incorporate the cluttered environmental conditions in which the animals interact with digital avatars.

## Technical Risks and Possible Solutions

The field of view of farm animal's eyes varies between species. For example, the typical field of vision for cattle is 330° while for pig it is 310°. Generally speaking, unlike humans' farm animals such as cows and pigs can prioritize lateral monocular vision and thereby increase the panoramic view while decreasing the bifocal vision. Hence, the deepfake technologies developed for humans cannot be easily translatable or adapted for livestock farming applications. To avoid compromising the viewing experience and to overcome the screen-door effect, the resolution limit of the animal's visual system, the visual angle and acuity factors should be considered in the designing of digital avatars. In addition to the field of view and efficiency, brightness, form factor, vergence accommodation conflict are additional technical challenges in the process of development of deepfake technologies as experienced in the human applications (64). Holographic projection has been suggested as a way to overcome the form factor in the augmented reality for human gaming applications (65). By manipulating the light field displays and the light fields along with the possibility of using contact lenses, the vergence accommodation problem can be overcome (66, 67). Developing digital avatars and deepfake technologies for livestock by deriving inspirations from human based solutions has to overcome anthropomorphism.

Regarding the accuracy, efficiency and added value that deepfake technologies can bring to livestock farming, it is important to highlight the extremely high quality of the real data that is required to train the models with. The model learning should be well-supervised and validated to ensure no wrong classification or labeling is created within the algorithm. Empirical evidence or studies within livestock farming is currently absent as GANs and their applications are still in their infant stages and have to date only been explored in a few scientific contexts.

## Summary

In conclusion, similar to all AI implementations, deepfakes also have positive and negative impacts. The potential positive effects of deepfakes are still new areas that are under exploration, and as such, it may require some time for these technical architectures to mature and being vastly implemented in the public domain. Their contribution to biomedical and behavioral applications, on top of agricultural practices, demonstrates that few of these applications might soon surface and help balance the adverse impacts of deepfakes. However, at higher stakes, various standardizations and security measures will be required, along with implementations of such technologies to ensure that no manipulations can take place. Pilot studies and explorative experiments are necessary to allow a better understanding of what deepfake technologies can mean for scientific purposes beyond us humans.

## Author Contributions

The author confirms being the sole contributor of this work and has approved it for publication.

## Conflict of Interest

The author declares that the research was conducted in the absence of any commercial or financial relationships that could be construed as a potential conflict of interest.

## Publisher's Note

All claims expressed in this article are solely those of the authors and do not necessarily represent those of their affiliated organizations, or those of the publisher, the editors and the reviewers. Any product that may be evaluated in this article, or claim that may be made by its manufacturer, is not guaranteed or endorsed by the publisher.
